# Supplementary material for: Depression symptoms among University Students in the Khulna region of Bangladesh
Source: PLOS Ment Health. 2025 Apr 16;2(4):e0000158. doi: 10.1371/journal.pmen.0000158 (PMC12798502; doi:10.1371/journal.pmen.0000158)
Supplement: Attachment — Submitted filename: Response to Reviewers comments.docx [file pmen.0000158.s003.docx]

**PMEN-D-24-00166**

**Depression symptoms among University Students in the Khulna region of Bangladesh**

We thank the editor and reviewers for their comments and appreciate the care taken in reviewing our manuscript. We hope that the revisions adequately address the concerns of the editor and reviewers. Below we provide our response to the editor’s and reviewers’ comments. All changes to the manuscript have been tracked in the file labeled ‘Revised Manuscript with Track Changes’.

| **Journal Requirements:** | | |
| --- | --- | --- |
|  | Comments | Responses |
| 1. | Please send a completed 'Competing Interests' statement; including any COIs declared by your co-authors. If you have no competing interests to declare, please state "The authors have declared that no competing interests exist". | We have clearly stated that all authors declared that they don’t have any competing interest. |
| 2. | Please amend your detailed Financial Disclosure statement. This is published with the article. It must therefore be completed in full sentences and contain the exact wording you wish to be published.  If you did not receive any funding for this study, please simply state: “The authors received no specific funding for this work.” | Added as suggested. |
| 3. | All PLOS journals now require all data underlying the findings described in their manuscript to be freely available to other researchers, either  1.In a public repository,  2.Within the manuscript itself, or  3. Uploaded as supplementary information. | Thanks for your concerned. We have  clearly stated under “Data availability  statement” that “The data used in this  Study can potentially be shareable upon appropriate request to the corresponding author”. |
| 4. | Please ensure that the Title in your manuscript file and the Title provided in your online submission form are the same. | We ensured that the tittle in manuscript file and the tittle provided in online submission form are the same. |
| **Reviewer 1** | | |
| 1. | Here are some of the recommendations:  1. Some statements have to be edited for grammar and completion.  2. Clearly operationalize the term/construct of depression explored in this study to avoid confusion.  3. You may include in the Introduction existing policies/programs/actions being implemented in the Khulna region that aim to mitigate depression.  4. In the Methods, what are the characteristics of these institutions? Why were they purposively chosen? Why only four institutions?  5. Based on the study's objective, the frequency (stated in the Abstract on the Cover Page, p. 1, but this is different from the Abstract on p. 5) of depression among university students was explored. How was this established by PHQ-9, considering that it only measures the participants’ depressive symptoms within two weeks? Kindly clarify.  6. Briefly describe your questionnaire for the demographics/other variables mentioned in your Results. | Thank you for your comment.  1. We have now edited statements for grammar and completion.  2. We have Clearly operationalized the term/construct of depression explored in this study to avoid confusion.  3. We have now added existing policies/programs/actions being implemented in the Khulna region that aim to mitigate depression.  4. In terms of methodology, the research focuses on universities in Bangladesh's Khulna area, and these establishments are defined as public universities. It was purposefully selected in order to guarantee that Diversity offered by four universities can contribute to a thorough grasp of the subject being studied at the same time; the sample is representative of the larger university landscape in the Khulna region, which may be essential for the research goals. In addition, accessibility and cooperative data-sharing policies. Because of timing and resource constraints the study narrowing down on a smaller group of institutions, which may examine each one in greater detail, enabling more thorough analysis and comprehensive data gathering. Simultaneously providing targeted, detailed insights to enhance the study's value.  Based on the study's objective, the frequency (stated in the Abstract on the Cover Page, p. 1, depression rate mentioned female students reporting heightened rates (71.7%) than male students (62%). but on p. 5 sample size mentioned The sample comprised 597 men (59.7%) and 403 women (40.3%). It is only measured since it is the simplest method and has been shown to be an effective means of identifying depression. Questionnaire for screening depression The answers are utilized to forecast an individual's level of depression and the situation in which he or she is at the time of the survey. In the PHQ-9 questionnaire, study participants were asked how often they were bothered by nine specific symptoms related to depression over the last 2 weeks where score ranging 1-4 indicates Minimal depression; 20-27 indicates Severe depression.  We have Briefly described questionnaire for the demographics/other variables mentioned in Results in line (206 -216) |
| **Reviewer 2** | | |
| 1. | Introduction: Page 6:  Lines 7-8, needs in-text citation  Line 14, spell out UK first, before abbreviating  Line 16, why single-out Kenya? This statement should be deepened, backed-out with other existing literature globally. Please revise/enhance the statement.  Line 18, you mention globally, and yet, you did not provide literature proof on this. Please include in-text citations on this statement.  Lines 20-27, in a unilateral fashion, you mentioned conducted research in different countries on depression. This presentation may be improved by being more inclusive of which countries you will name or include. You also mentioned ‘in various European countries’ (line 22) but did not enumerate in the following statements whichever countries you were referring to. In your reference list, there is only one author, so how can you say Various?  Lines 28-29, continuing to Page 7, lines 1-2, you talk about a number of global studies, but your references are only from two authors (Koly et al., 2020 and Akhtarul et al., 2020) who did research in Bangladesh. How is this global? Also, this is a two-sentence paragraph, please improve/revise this.  Page 7:  Lines 3-16, Please strengthen your context, argument, justification and contribution. Your paragraph, although bearing how you give importance to mental health problems, does not give a clear picture of why among university students in the Khulna region of Bangladesh.  General Comment: What is your hypothesis? What is the main objective of this cross-sectional study? Please include in your Introduction section | Thank you for your comment. We have now added the additional information, strengthen context, argument, justification and contribution the introduction section and clarified the objective of this study in line (54- 125). |
| 2. | Methodology: General Comment for Methodology: Before discussing the setting and population, please give a brief discussion on your Research Method. Instead of just mentioning that this is a cross-sectional study, include a sub-section, Research Method, and discuss what design/method you utilized and why the choice of this?  Page 7:  Line 20, you mentioned ‘purposively selected’ but you did not include the parameters of your research. Please include your inclusion and exclusion criteria. Also, explain what random walk technique is.  Line 24, if you randomly selected, what was your recruitment/sampling technique? Please include this in the discussion  General comment for Setting and population: Please separate your discussion on your data-collection procedure from the setting and population.  Page 8:  Line 10, introduce first what is PHQ-9, and why you chose this for your research instrument. Give a background of this assessment tool, mention the validity and reliability and how you were able to access this.  Lines 22-29, to give better context on your statistical analysis, please start your presentation of this by recalling the objective of your study.  Page 10:  Line 2, give the year of authorship for Godden | Thank you for your comment. Research Method, design/method and why the choice of this have added in line (128-137).  Line 133-137, The rationale for purposive selection mentioned. Line 141-145, inclusion and exclusion criteria included.  Line 154- 157 we have discussed.  Line 165-171 we have separate data-collection procedure from the setting and population.  Line 172-188, we have described PHQ-9 and a background of this assessment tool described.  Lines 189-197 we have given a better context and started presentation of this by recalling the objective of study.  159-160 we have given the year of authorship for Godden |
| 4. | Results  General Comments:  Your Results section must be presented in accordance to the objectives of this research and not according to the Statistical Tools and Treatment. Since you were not able to clearly state the overall aim of this research, the presentation of your results in not well-presented. Please revise accordingly.  Page 12, Table 1. Please improve your table presentation. Follow the correct table formatting. | Thank you for your comment. We have worked to make the Results section engaging and interesting. |
| 5. | Discussion: Your first paragraph here should be aligned with your presentation of results. The discussion section must be parallel to your Results.  Page 16:  Lines 8-9, you mentioned about a gender angle, however, you failed to closely contextualize this with cultural aspects.  Lines 11-12, this should be cross-referenced  Line 19, you presented the finding, but you did not offer an attribution or contextualization  General Comments on Discussion:  As a general guide, please evaluate your presentation, making sure that the importance and relevance of your findings are captured, contextualized and that you have deepened the interpretation of the results in relation to your research objectives and literature review.  Page 18:  Please transfer Strengths and Limitations as a sub-section of Conclusion. Also, include in your discussion, how you were able to manage the limitations of your study and how you maximized the strengths for the success of this research. | We have addressed this factor and aimed to provide a more balanced discussion. Additionally, we included new information about a gender angle.  Line 355-385 we have included how we were able to manage the limitations of your study and how you maximized the strengths for the success of this research. |
| 6. | Conclusion  General Comments:  Please enrich your conclusion. This must be aligned with your results and discussion. Include implications to theory and practice as well as future directions. | Line 387-412 we have enriched conclusion aligned with your results and discussion. |
