# Supplementary material for: Depression symptoms among University Students in the Khulna region of Bangladesh
Source: PLOS Ment Health. 2025 Apr 16;2(4):e0000158. doi: 10.1371/journal.pmen.0000158 (PMC12798502; doi:10.1371/journal.pmen.0000158)
Supplement: Attachment — Submitted filename: Response_to_Reviewers_comments_auresp_2.docx [file pmen.0000158.s004.docx]

**PMEN-D-24-00166R1**

**Depression symptoms among University Students in the Khulna region of Bangladesh**

We thank the editor and reviewers for their comments and appreciate the care taken in reviewing our manuscript. We hope that the revisions adequately address the concerns of the editor and reviewers. Below we provide our response to the editor’s and reviewers’ comments. All changes to the manuscript have been tracked in the file labeled ‘Revised Manuscript with Track Changes’.

| **Journal Requirements:** | | |
| --- | --- | --- |
|  | Comments | Responses |
| 1. | Please review your reference list to ensure that it is complete and correct. If you have cited papers that have been retracted, please include the rationale for doing so in the manuscript text, or remove these references and replace them with relevant current references. Any changes to the reference list should be mentioned in the rebuttal letter that accompanies your revised manuscript. If you need to cite a retracted article, indicate the article’s retracted status in the References list and also include a citation and full reference for the retraction notice. | Thanks for your concerned. We ensured that the reference list is complete and correct. The reference list mentioned in line (445-643). |
| **Reviewer 2** | | |
| 1. | For lines 157-158, introduce your table. Place the Table number and description before the actual Table. Improve the presentation of your table showing the sample. | Thank you for your comment. We have now added the Table number and description before the actual Table and Improve the presentation of your table showing the sample in line (158-159). |
| 2. | For lines 159-166. Introduce first your figure. Place the Figure number and description before the actual Figure. Improve the presentation of your figure showing the formula. | Thank you for your comment. Line (160-170) We have worked to improve the presentation of figure showing the formula. |
| 3. | For lines 203-204. Place the Figure number and description before the actual Figure. Improve the presentation of your figure showing the map. | Thank you for your comment. We have placed the Figure number and description before the actual Figure mentioned in line (204). |
| 4. | For line 218. Improve your result sub-title, Change Chi-square test to something more specific, like what did this statistical treatment measure? | Thank you for your comment. We have Improve your result sub-title, Change Chi-square test to specific and also clarified statistical treatment measure in line (210). |
| 5. | For line 247. Improve your result sub-title, Change Binary logistic regression to something more specific, like what did this statistical treatment measure? | We have Improve your result sub-title, Change Binary logistic regression to specific in the line (254-255). |
| 6. | For lines 270-271. This table is adjusted to Table 2 already, since Table 1 is the sample presentation | Thank you for your comment. We have now adjusted to Table 2 already, since Table 1 is the sample presentation in line (210,254-255). |
| 7. | For lines 272-274. This table is adjusted to Table 3 already, since Table 2 is the Frequency and Association of Factor Categories with Depression Symptoms. | Thank you for your comment. We have now additional information related to table. |
| 8. | For lines 372-387. Please move Strengths and Limitations after your Conclusion | Thank you for your comment. In line (414-429) we moved Strengths and Limitations after Conclusion. |
